# HLA Haplotype Mismatch Transplants and Posttransplant Cyclophosphamide

**DOI:** 10.1155/2016/7802967

**Published:** 2016-04-07

**Authors:** Andrea Bacigalupo, Simona Sica

**Affiliations:** Istituto di Ematologia, Universita' Cattolica del Sacro Cuore, Fondazione Policlinico Universitario Gemelli, Rome, Italy

## Abstract

The use of high dose posttransplant cyclophosphamide (PT-CY) introduced by the Baltimore group approximately 10 years ago has been rapidly adopted worldwide and is becoming a standard for patients undergoing unmanipulated haploidentical (HAPLO) transplants. PT-CY has been used following nonmyeloablative as well as myeloablative conditioning regimens, for bone marrow or peripheral blood grafts, for patients with malignant and nonmalignant disorders. Retrospective comparisons of HAPLO grafts with conventional sibling and unrelated donor grafts have been published and suggest comparable outcome. The current questions to be answered include the use of PT-CY for sibling and unrelated donors transplant, possibly in the context of prospective randomized trial.

## 1. HLA Haplotypes and Haploidentical Transplants

The short arm of chromosome 6 is home to the human major histocompatibility complex (MHC) genes, which code for human leukocyte antigens (HLA): during meiosis, the MHC region does not undergo “crossing over” (except for rare events), and we inherit one HLA haplotype from the father and one from the mother. If we name A/B and C/D the four parental haplotypes, the offspring can be A/C, B/C, A/D, or B/D. Mother and son are therefore called haploidentical (we will use HAPLO in this review), meaning that donor and recipient genetically share 1 HLA haplotype, not only the 5 HLA loci, but all the genes in the MHC; of course, mother and son will be mismatched on the other HLA haplotype; a brother can be a HAPLO donor but also an uncle or a cousin. When it comes to HLA antigens, if we consider the 5 HLA loci A, B, C, DRB1, and DQ, a HAPLO donor should share 5/10 HLA antigens; however, because parental HLA haplotypes may share antigens, mother and son may share more than 5/10 HLA antigens. However, there is no evidence that a 5/10 HLA matched or a 7/10 HLA matched HAPLO donor will produce significantly different outcome [1].

## 2. Biology of HAPLO Transplants

The double problem of a HAPLO transplant is rejection of the graft, or host versus graft (HvG), and rejection of the host, or graft versus host disease (GvHD), and this double problem was the cause of failure of initial attempts. The first successful haplotype mismatch transplants were carried out in the early 1980s in severe combined immunodeficiency (SCID) patients, in whom the risk of rejection is minimal [2]: GvHD was prevented using T cell depletion (TCD) in the absence of any additional posttransplant GvHD prophylaxis [2]. The program of TCD HAPLO transplants was expanded to leukemia patients, in the nineties, with the advent of G-CSF mobilized peripheral blood (PB), as a stem cell source, and the use of CD34 selection, as a method of TCD [3]. Despite encouraging long term results, mortality due to infections remained high, 30% or higher, as a consequence of prolonged immune deficiency, caused by removal of T cells from the stem cell source [3]. Immune recovery has become more rapid with the use of alfa-beta T cell and CD19 B cell depletion [4].

In the last decade, Wang and coworkers have shown that HAPLO transplants can be performed without T cell depletion, with intensive immune depletion with antithymocyte globulin (ATG), cyclosporine a (CsA), methotrexate (MTX), and mycophenolate (MMF) [5], and these are referred to as T cell replete HAPLO grafts: toxicity was acceptable and long term results are not much different when compared to matched sibling donor grafts. An alternative way of preventing GvHD in HAPLO transplants is to use high dose posttransplant cyclophosphamide (PT-CY) [6]: the present review will concentrate on the latter modality.

## 3. Unmanipulated Marrow Grafts following a Nonmyeloablative Conditioning Regimen (NMA) and PT-CY

The John Hopkins group hypothesized 40 years ago that high dose cyclophosphamide (CY), given 3 days after infusion of mismatched grafts, would protect rats from graft versus host disease (GvHD) [7]: this would occur because alloreactive T cells would be in the *S* phase on day +3 and would be killed by a large dose of CY (50 mg/kg). This phenomenon of* “posttransplant purging”* would spare T cells not undergoing active proliferation, which may include pathogen specific T cells and hopefully some T cells exerting a graft versus leukemia effect (GvL). This hypothesis was brought to the clinic in a study reported in 2008 [6]: a group of patients received unmanipulated marrow following a nonmyeloablative conditioning (NMA) regimen of low dose CY, fludarabine (FLU), and TBI 2 Gy; CY 50 mg/kg was given on days +3 and +4, with tacrolimus (TAC) and mycophenolate (MMF) starting on day +5 [6]. Engraftment was achieved in 87% of patients at a median interval from transplant of day +16 for neutrophils and day +24 for platelets; acute GvHD, grades II–IV and III-IV, was, respectively, 34% and 6% and chronic GvHD was 4%. Nonrelapse mortality (NRM) was less than 20%, though relapse was significant (60%). The overall actuarial survival was in 40% range for lymphoid and 20% for myeloid malignancies. This study proved that posttransplant CY (PT-CY) was feasible, prevented GvHD, and allowed engraftment, although relapse was high. The experience at John Hopkins has been recently updated [8] on 372 patients, receiving the same NMA regimen originally described, bone marrow as a stem cell source and PT-CY, tacrolimus, and mycophenolate for GvHD prophylaxis. The 6-month nonrelapse mortality is 8%; the 4-year probability of relapse and survival is, respectively, 46% and 50%. The disease risk index (DRI) was the strongest predictor of survival: 71%, 48%, and 35% for low, intermediate, and high risk patients [8]. This study would suggest that disease burden dictates the fate of the transplant, and it is known that patients with a high load of leukemia require more intensive conditioning regimens.

## 4. Unmanipulated Marrow Grafts following a Myeloablative (MA) Conditioning Regimen

A study using PT-CY and myeloablative conditioning (MAC) regimens has shown a low incidence of graft failure (<5%), a low incidence of acute grades II–IV GvHD (18%), a very low rate of severe grades III-IV acute GvHD (3%), and relapse as expected with a conventional CyA+MTX GvHD prophylaxis [9]. This study included 2 variations of the protocol: CyA was started on day 0 and MMF on day +1 (before PT-CY), and PT-CY was given on days +3 and +5 (rather than +3+4). An update on 148 patients, allografted from haploidentical family members, following MA regimen in the San Martino Unit in Genova, has been recently published [10]: all patients received a myeloablative regimen with posttransplant cyclophosphamide (PT-CY), between August 2010 and January 2014. A total of 63 patients had active disease at transplant; 46 were in first remission (CR1) and 39 in second remission (CR2), hematologic remission. The most common diagnosis was acute leukemia (*n* = 76), 48 cases of acute myeloid leukemia (AML) (27% with active disease), and 24 cases of acute lymphoblastic leukemia (ALL) (32% with active disease). Most of the patients with non-Hodgkin lymphoma (60%) also had advanced disease, as well as patients with myelofibrosis. The median age of the entire group was 47 years range 17–74. The 4-year cumulative incidence of NRM was 14%. It was not predicted by patients' age: 8%, 18%, and 17% for patients aged <40 (*n* = 51), 41–60 (*n* = 62), and over 60 (*n* = 35) (*p* = 0.2). It was also not predicted by disease phase: 9%, 15%, and 17% for patients in CR1 (*n* = 46), CR2 (*n* = 39), and advanced disease (*n* = 63) (*p* = 0.4). The cumulative incidence of relapse related death (RRD) was 27% and could be predicted by disease phase: 11% for CR1 patients, 26% for CR2 patients, and 40% for patients with active disease at the time of transplant (*p* = 0.003) [9]. With a median follow-up of over 430 days, the actuarial overall survival is 77% for CR1 patients and 49% and 38% for CR2 and advanced patients (*p* = 0.0001) [10].

## 5. Unmanipulated Peripheral Blood Grafts and PT-CY

Several centers have elected to use exclusively peripheral blood (PB) as a stem cell source in place of bone marrow (BM), based on donor preference, inability to secure operating room hours, and outcome considerations. These centers are unwilling to use BM and have thus adopted the Baltimore protocol using unmanipulated PB instead of BM. Results have been unexpectedly encouraging: this is clear when PB is given after the original Baltimore NMA conditioning regimen. In a recent paper, unmanipulated BM and PB have been compared in the NMA setting: incidence of acute and chronic GvHD, nonrelapse mortality, relapse, and survival were quite comparable [11]. Things may be a little bit more complicated when unmanipulated PB is given after MA regimen, such as full dose TBI: in the Atlanta program with TBI 12 Gy, GvHD, grades III-IV, has been reported to be 23% and the cumulative incidence of chronic GvHD is 56%, (severe in 10%); however, overall NRM was 3% and 0% for patients with early intermediate risk disease [12]. The Atlanta group has also reported a busulfan based regimen (BU 110–130 mg/m^2^ on each of the 4 days) + FLU and CY, with a very low NRM of 10% and a disease-free survival of 60% [13]. These results are excellent and suggest that unmanipulated PB can be given in the context of a HAPLO donor and PT-CY.

The transplant group in Milano has combined the use of PT-CY with rapamycin (RAPA) and MMF, following HAPLO mismatched PB grafts [14]: acute GvHD, grades II–IV and III-IV, was 15% and 7,5%, respectively, and chronic GvHD was 20%; NRM at 1 year was 17%, with a disease-free survival at 1 year of 71% for remission patients.

## 6. The Two-Step Approach of PT-CY

One group has devised a so-called two-step approach [15]: patients receive a conventional dose of total body irradiation (12 Gy) over 4 days and then a high dose of donor lymphocytes (2 × 10^8^/kg), followed after 72 hours by CY 50 mg/kgx2, followed by tacrolimus + mycophenolate on day −1. Finally, on day zero, patients receive CD34 selected cells, from G-mobilized peripheral blood [14]. Results, though only in patients in hematologic remission, were very good with a 2-year nonrelapse mortality of 3.6% and a disease-free survival of 74% [14].

## 7. Regimens other than PT-CY for Unmanipulated HAPLO Transplants

**Figure 1:**
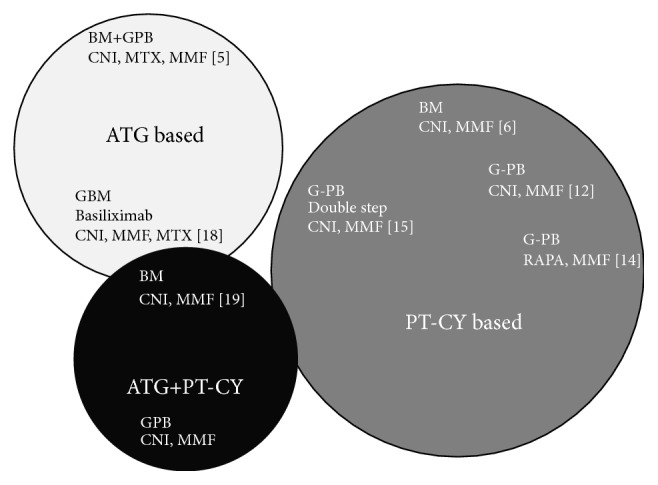
Different platforms of GvHD prophylaxis for patients undergoing a haploidentical stem cell transplant.* ATG: programs based on antithymocyte globulin (ATG)* include the original Chinese protocol [5] using bone marrow (BM) + G-mobilized peripheral blood (G-PG) and cyclosporin (CsA), mycophenolate (MMF), and methotrexate (MTX). Also it includes the modified G-mobilized BM (G-BM) + CsA + MMF + MTX + basiliximab [18].* PT-CY: programs based on posttransplant cyclophosphamide (PT-CY)*. The original Baltimore protocol [6], with unmanipulated BM, a calcineurin inhibitor (CNI), and MMF. The modified program with rapamycin and G-PB [12] and the double step approach [14]* ATG + PT-CY*.* The combined use of ATG + PT-CY* as proposed by the Baltimore group in sickle cell disease [19], or by the French cooperative group using G-PB as a stem cell source (unpublished).

There are basically 3 GvHD prophylaxis regimens for unmanipulated HAPLO stem cell transplants (Figure 1).

### 7.1. ATG Based

The first, also in time, is the Chinese antithymocyte (ATG) based regimen, first published in 2006 [5] combining ATG, CyA, MMF, and MTX and updated in 2013 [16]. This regimen has been modified to include basiliximab and G-mobilized BM [17, 18] (Figure 1). The ATG based programs have enrolled a rather large number of patients, with follow-up now over 10 years, in the context of MA conditioning regimen: acute GHD grades III-IV is reported to range between 5% with BM and 17% with PB (Table 1). Chronic GvHD is 17% for BM and up to 53% for PB (Table 1), and NRM is 36% for G-mobilized BM, versus 18/30% for grafts including PB in the stem cell source (Table 1).

### 7.2. PT-CY Based

The second approach is PT-CY (Figure 1): Table 1 summarizes three reports using either PB or BM following MA regimen and PT-CY. Acute GvHD, grades III-IV, is more frequent when PB is used as a stem cell source, as well as chronic GvHD. NRM is very low in patients receiving BM or G-PB.

### 7.3. ATG + PT-CY

Finally, a third platform exists, the combination of ATG and PT-CY (Figure 1). This approach has been used in Baltimore for patients with sickle cell disease (SCD): the rationale is to ensure engraftment on the one hand and on the other hand avoid acute and chronic GvHD altogether in a nonmalignant disorder [19]; 17 patients were grafted following the Baltimore NMA regimen with the addition of ATG upfront, all 17 survive, and no patient developed acute or chronic GvHD [19]; 11/17 (65%) are free of SCD. A similar protocol is being adopted for patients with thalassemia. The French cooperative group is launching a trial on the combined use of ATG and PT-CY, with the aim of reducing the risk of acute and chronic GvHD in patients receiving unmanipulated PB grafts from HAPLO identical family donors. Early results would suggest a strong protection against GvHD. We shall see what the combination of ATG and PT-CY will do in patients with malignant disorders, in terms of control of the original disease.

For the time being an analysis of the EBMT is underway to assess, in retrospect, but in a large number of patients, the outcome of ATG based or PT-CY protocols in unmanipulated HAPLO grafts.

## 8. Comparison of HAPLO with PT-CY and Unrelated Donor Transplants

A recent CIBMTR study has compared the outcome of HAPLO transplants for acute myeloid leukemia (AML) with PT-CY (*n* = 192), with 8/8 matched unrelated donor grafts (*n* = 1982) [20]. Patients, grafted between 2008 and 2012, were stratified according to myeloablative (MA) or reduced intensity (RiC) conditioning regimens. Recipients of haploidentical transplantation (mismatched at least two or more HLA loci to donors) received an unmanipulated, predominantly BM, graft with GvHD prophylaxis consisting of posttransplant cyclophosphamide, CNI, and mycophenolate mofetil. Recipients of unrelated transplantation (matched at the allele level at HLA-A, -B, -C, and -DRB1) received predominantly unmanipulated peripheral blood and GvHD prophylaxis consisting of a CNI and methotrexate or mycophenolate mofetil. The conclusions of this study are as follows: no differences in overall and leukemia-free after MUD transplant as compared to HAPLO; lower relapse after reduced intensity conditioning, negated by higher NRM after MUD transplant; no differences in relapse/NRM risks after myeloablative MUD transplant; higher chronic GvHD after MUD transplant regardless of intensity of conditioning regimen; and higher grades 2–4 acute GvHD with only myeloablative regimen [20]. Although statistical power is limited, these data suggests that survival for patients with AML after haploidentical transplantation with PT-CY is comparable with matched unrelated donor transplantation.

Other studies have shown superimposable outcome of transplants from HAPLO compared to unrelated and also sibling donors [13, 21–26]. The lack of prospective randomized trials makes it difficult to prioritize a given donor type; however, HAPLO transplants offer several advantages, including the high probability of finding a donor, a short time to transplant, low cost, and comparable outcome. For these reasons, there have been recently more HAPLO as compared to cord blood transplants [27].

## 9. Conclusions

Unmanipulated HAPLO transplants are rapidly increasing worldwide, indicating encouraging results and reproducible outcome. GvHD and NRM are acceptable. Disease-free survival appears to be comparable to transplants from sibling or unrelated donors.

Several questions remain to be answered: what is the best dose and timing of PT-CY? Should we use ATG or PT-CY based GvHD prophylaxis? Should we use a combination of both for best protection against GvHD? Should we be using the same GvHD prophylaxis also for unrelated donor transplants, in which we still see significant acute and chronic GvHD? Is bone marrow the best stem cell source, or can we also use unmanipulated G-mobilized peripheral blood? And, above all, is the increasing use of HAPLO transplants going to change the algorithm for donor selection? As usual, it will take several years to answer some of these questions. However, one fact has already been ascertained: HAPLO transplants have made the procedure available for almost 100% of eligible patients, and this is clearly a very significant improvement.

## Figures and Tables

**Table 1:** Outcome of different GvHD prophylaxis regimens and different SC sources, in the context of a myeloablative conditioning regimen.

SC	Age	Phase of disease	*N*	aGvHD III-IV	cGvHD mod-sev	NRM	REF
ATG based							
BM + G-PB	25 y	CR + Ad	756	14%	53%	18%	[16]
G-PB	25 y	CR	99	17%	41%	30%	[25]
G-BM	37 y	CR + Ad	80	5%	17%	36%	[18]
PT-CY based							
G-PB	46 y	CR	30	23%	27%	3%	[12]
G-PB	55 y	CR + Ad	40	7,5%	20%	17%	[14]
BM	47 y	CR + Ad	148	3%	16%	14%	[10]

CR + Ad: patients in complete remission and patients with advanced disease; SC: stem cell source; BM: bone marrow; G-PB: peripheral blood mobilized with GCSF; G-BM: BM mobilized with GCSF; aGvHD: acute GvHD, grades III-IV; cGvHD: chronic GvHD, moderate to severe; NRM: nonrelapse mortality; REF: reference number.

ATG: antithymocyte globulin; PT-CY: posttransplant cyclophosphamide.
